# 4476 Association between socioeconomic status and comorbid conditions in a population of diabetes patients

**DOI:** 10.1017/cts.2020.107

**Published:** 2020-07-29

**Authors:** Riza Li, Kevin Ndura, Claudine Jurkovitz

**Affiliations:** 1 University of Delaware: DE-CTR ACCEL; 2 Christiana Care Health System

## Abstract

OBJECTIVES/GOALS: To reduce hospitalizations, health care systems are studying ways of improving social determinants of health (SDoH) in patients with chronic disease such as diabetes (DM). Our goal was to better characterize the SDoH of a cohort of DM patients by using socio-economic information from census data. METHODS/STUDY POPULATION: Our study population included DM patients seen in primary care practices of a large health care system in 2013-2017. We integrated socio-economic status (SES) information from the American Factfinder to data extracted from the electronic health record (EHR). Addresses for the cohort were geocoded using ArcMap to obtain the census tract information for median income, poverty status, educational level, and supplemental food benefits using American Community Survey 5-Year estimates. We used multivariable logistic regression to calculate odds ratio (OR) and 95% confidence intervals [], with 3+ comorbidities as the dependent variable and demographic and SES variables as independent variables. RESULTS/ANTICIPATED RESULTS: Our study population included 13,782 patients: 53% were female, 65% white, 28% Black, 27% were on Medicare, 3% on Medicaid, median age was 60, 53% had 3+ comorbidities. Median income was $66,243, poverty level 6%, receiving food benefits 8%, no high school degree 8%, and bachelor’s degree or higher 30%. After evaluating collinearity, our multivariable analysis showed that patients with 3+ comorbidities were more likely to have income < $52,000 (lower quartile) versus $84,001 (upper quartile), OR = 1.2 [1.0-1.4]; be female, OR = 1.6 [1.4-1.7]; divorced or widowed versus married, OR = 1.5 [1.3-1.7], 1.4 [1.3-1.6]; and be on Medicare, Medicaid or both, OR = 2.4 [2.2-2.6], 2.2 [1.8-2.6], 6.0 [4.5-8.3]. DISCUSSION/SIGNIFICANCE OF IMPACT: Census tract-based SES could provide invaluable information to health care providers when associated to the EHR. We found that median income, which is not collected in the EHR, was significantly associated with a higher burden of disease. Census tract SES could serve as a proxy for evaluating SDoH.

